# Role of ACE2 in pregnancy and potential implications for COVID-19 susceptibility

**DOI:** 10.1042/CS20210284

**Published:** 2021-08-02

**Authors:** Nayara Azinheira Nobrega Cruz, Danielle Stoll, Dulce Elena Casarini, Mariane Bertagnolli

**Affiliations:** 1 Department of Medicine, Discipline of Nephrology, Federal University of Sao Paulo, São Paulo, Brazil; 2 Research Center of the Hospital Sacré-Coeur, CIUSSS Nord-de-l’Île-de-Montréal, Montréal, Canada; 3 School of Physical and Occupational Therapy, Faculty of Medicine, McGill University, Montréal, Canada

**Keywords:** angiotensin converting enzyme 2, cardiovascular disease, COVID-19, placental biology, pregnancy, SARS-COV-2

## Abstract

In times of coronavirus disease 2019 (COVID-19), the impact of severe acute respiratory syndrome (SARS)-coronavirus (CoV)-2 infection on pregnancy is still unclear. The presence of angiotensin-converting enzyme (ACE) 2 (ACE2), the main receptor for SARS-CoV-2, in human placentas indicates that this organ can be vulnerable for viral infection during pregnancy. However, for this to happen, additional molecular processes are critical to allow viral entry in cells, its replication and disease manifestation, particularly in the placenta and/or feto–maternal circulation. Beyond the risk of vertical transmission, COVID-19 is also proposed to deplete ACE2 protein and its biological actions in the placenta. It is postulated that such effects may impair essential processes during placentation and maternal hemodynamic adaptations in COVID-19 pregnancy, features also observed in several disorders of pregnancy. This review gathers information indicating risks and protective features related to ACE2 changes in COVID-19 pregnancies. First, we describe the mechanisms of SARS-CoV-2 infection having ACE2 as a main entry door and current evidence of viral infection in the placenta. Further, we discuss the central role of ACE2 in physiological systems such as the renin–angiotensin system (RAS) and the kallikrein–kinin system (KKS), both active during placentation and hemodynamic adaptations of pregnancy. Significant knowledge gaps are also identified and should be urgently filled to better understand the fate of ACE2 in COVID-19 pregnancies and the potential associated risks. Emerging knowledge will be able to improve the early stratification of high-risk pregnancies with COVID-19 exposure as well as to guide better management and follow-up of these mothers and their children.

## Introduction

The world is facing one of the greatest pandemics in history. The coronavirus disease 2019 (COVID-19) is a respiratory infection caused by the newly discovered severe acute respiratory syndrome coronavirus 2 (SARS-CoV-2), which emerged in Wuhan, China, in December 2019 [1]. The SARS-CoV-2 is closely related to SARS-CoV and shares similarities with the Middle East Respiratory Syndrome (MERS)-CoV, both responsible for severe respiratory infection outbreaks in 2002 and 2012, respectively [2,3].

Pregnant women are particularly susceptible to respiratory infections. Pregnancy naturally involves immunological changes that can increase susceptibility to pathogens. Key cardiopulmonary adaptations of pregnancy include diaphragm elevation, increased oxygen consumption, and edema of the respiratory tract, rendering pregnant women more prone to hypoxia [4–6]. Indeed, during previous coronavirus (CoV) pandemics approximately 50% of pregnant women with SARS-CoV and 44% of those with MERS-CoV required intensive care [5,7]. In both outbreaks, approximately 40% of the affected pregnant women needed mechanical ventilation and the mortality rate reached up to 25%.

The impact of COVID-19 on pregnant women’s health and on fetal development is still unknown. To this date, COVID-19 is described as less severe in pregnant women than SARS-CoV or MERS-CoV, with a significant lower mortality rate [7–10]. However, pregnant women are more prone to develop severe manifestations of COVID-19 [11–13]. In addition, the broad and rapid transmissibility of SARS-CoV-2 compared with other coronaviruses makes this disease even more threatening. It also follows that the discovery of more dangerous variants and the report of more severe disease manifestations in individuals with cardiovascular and metabolic pre-conditions makes pregnant women with obesity, diabetes, or hypertension more vulnerable to COVID-19 [12–15].

Another important aspect rendering pregnant women more susceptible to COVID-19 is the fact that SARS-CoV-2 uses the protein angiotensin-converting enzyme (ACE) 2 (ACE2) as receptor to invade cells [16,17]. This characteristic of COVID-19 can negatively affect maternal and fetal health in many ways. ACE2 regulates the renin–angiotensin system (RAS) by converting angiotensin (Ang) I and Ang II into Ang 1–9 and Ang 1–7, respectively [18,19]. ACE2 is also largely expressed in the placenta and fetus throughout gestation, turning the placenta into a potential target for SARS-CoV-2 infection and for vertical transmission to the fetus [20–22]. Nevertheless, vertical transmission is still debated, since some placentas lack a colocalization between ACE2 and the transmembrane serine protease 2 (TMPRSS2), a protease that is also essential for SARS-CoV-2 entry and replication in the cells [22–24].

The SARS-CoV-2/ACE2 complex is now well described and shows ACE2 degradation following cell entry [25]. The impact of such degradation on physiological systems regulated by ACE2 activity can be critical during pregnancy. ACE2 is dynamically modulated throughout gestation along with alterations in the RAS and kallikrein–kinin system (KKS), two hormonal systems in which ACE2 constitutes an important cross-link and regulatory component [20,26]. By regulating both systems, ACE2 actively participates in maternal hemodynamic adaptations and in placentation, hinting at a critical role by promoting healthy pregnancy. Inversely, lower ACE2 expression during pregnancy has been associated with preeclampsia and intrauterine growth restriction (IUGR) in humans and various animal models [20,27–30]. Therefore, ACE2 depletion caused by COVID-19 may become a risk factor for impaired placental vascularization, maternal blood pressure elevation and adverse pregnancy outcomes associated with placental dysfunction and also aggravate pre-existing conditions in pregnant women.

Taking into account all these important gaps and unresolved questions concerning the impact of COVID-19 in pregnancy and the role of ACE2, this review discusses evidence that ACE2 may paradoxically act as a risk factor for viral transmission but also as a protective factor by preserving physiological adaptations in COVID-19-exposed pregnancies. Here, we describe the mechanism of viral infection and how the placenta may be susceptible, or at some level protected, against this specific virus. Then, we review the main physiological roles of ACE2 in pregnancy, notably on maternal and placental hemodynamics and on placentation and by promoting physiological cardiac and renal adaptations in the mother during pregnancy. Finally, we summarize the current knowledge linking ACE2 and pregnancy disorders and how this knowledge can contribute to better understanding of maternal–child susceptibility resulting from COVID-19 infection during pregnancy.

## SARS-CoV-2: mechanism of infection

**Figure 1:**
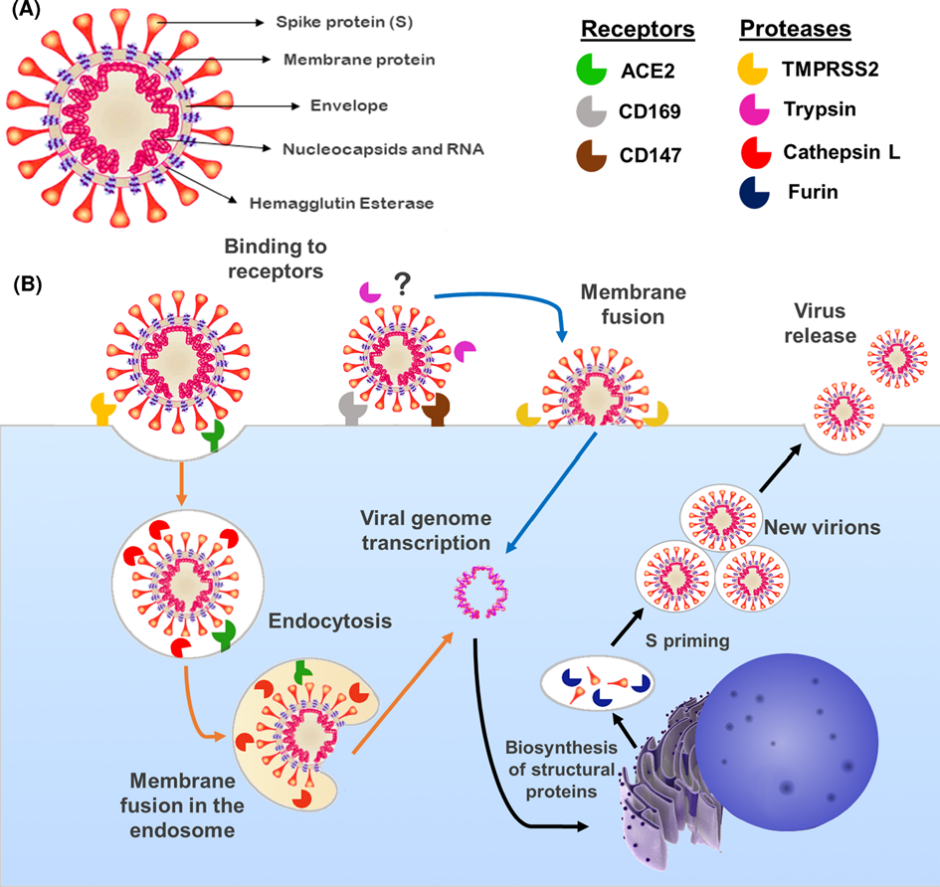
Structure of SARS-CoV-2 and proposed mechanisms of cell entry and replication in placental cells (**A**) SARS-CoV-2 is composed of RNA involved in nucleocapsids; the envelope retains the genetic material and contains the membrane protein and hemagglutinin–esterase dimers, attached to the envelope where the S protein responsible for receptor binding is located. (**B**) Proposed viral cell entry mechanisms in placental cells. SARS-CoV-2 binds to the transmembrane protein ACE2 or to alternative receptors such as CD147 and CD169 expressed in placental cells. Two fusion routes, the plasma membrane route (blue arrows) and the endosomal route (orange arrows), involve the S protein cleavage by TMPRSS2. Decreased pH inside the endosome can activate the protease cathepsin L, which can cleave S1, promoting the fusion of the SARS-CoV-2 with the endosome membrane and the release of the viral genetic material into the cytosol. After reaching the cytosol, both pathways have common steps (black arrows) with RNA transcription and replication and biosynthesis of viral proteins in the endoplasmic reticulum and Golgi apparatus. At this point, furin can prime S at S1/S2 before its release, potentiating the action of TMPRSS2 when the new virus is released to the extracellular environment.

SARS-CoV-2 is a single, positive-strand RNA virus belonging to the betacoronavirus B lineage. Structurally, SARS-CoV-2 comprises a spike protein (S), a membrane protein, an envelope protein, nucleocapsids, hemagglutinin–esterase dimers and its genetic material [3]. A graphical representation of the virus is presented in Figure 1A. The S is a transmembrane glycoprotein that can be cleaved into subunits S1 and S2. The S1 subunit contains the receptor-binding domain (RBD), which is the most variable part of coronaviruses’ genome and the segment responsible for SARS-CoV-2’s high affinity for human ACE2. Therefore, ACE2 works as a receptor allowing viral entry into cells [16,17,31]. A proteolytic activation of S is also crucial for membrane fusion [32]. There are two cleavage points in the S protein, one found between S1/S2 and another in S2, which can be cleaved by multiple proteases, such as furin, cathepsin and trypsin, but mostly by TMPRSS2 [3,16,31].

Two pathways are described for viral fusion in the cell membrane, both illustrated in Figure 1B. The entry through the plasmatic membrane route is possible if transmembrane proteases such as TMPRSS2 are present to cleave the S protein [16]. Alternatively, S1/S2 can be cleaved after the binding of the S protein with ACE2 by TMPRSS2 or other proteases, activating a second entry pathway where the virus is internalized via endocytosis [33]. Within the endosome, cathepsin L can be activated by low pH and alternatively cleave S in the S2 cleavage site, triggering the fusion of the virus with the endosomal membrane [33]. Independent of the pathway, once the viral genome reaches the cytosol, copies of the virus genome are transcribed and structural proteins are then synthesized [3,33].

In conclusion, the high affinity of SARS-CoV-2 to ACE2 in addition to the wide distribution of ACE2 and TMPRSS2 or other proteases involved in S priming, such as furin and cathepsin L, may define the high SARS-CoV-2 tropism and systemic clinical manifestation of COVID-19, varying from subclinical symptoms to multiple organ damage [3,16,34,35].

## Placental susceptibility to SARS-CoV-2 infection

Placental infection by viruses has several consequences for mother and fetus; hence, transplacental vertical transmission is a major concern. Vertical transmission can occur by intrauterine, intrapartum and postpartum mechanisms. Fortunately, most of the viral infections affecting the mother are not vertically transmissible [36]. However, even viral infections that affect only the placenta can compromise its function and promote pregnancy complications such as miscarriages, IUGR and preterm birth [36,37].

Initially, there was no confirmation of SARS-CoV-2 vertical transmission. However, as the virus spread globally, new cases of neonatal infection suggest vertical transmission [38]. In a more recent meta-analysis, 8.8% of neonates had a positive PCR or serological test indicating SARS-CoV-2 infection [39]. Furthermore, a quarter of the babies born from COVID-19 positive mothers developed symptoms including fever, tachypnea, shortness of breath and vomiting [39]. Nevertheless, because of the high incidence of preterm birth among COVID-19 positive mothers, it is difficult to determine if the symptoms are a consequence of prematurity-related complications or directly caused by SARS-CoV-2 infection, even though the symptoms appear more often among babies with COVID-19 [39].

There are also several limitations to the methods used to detect vertical transmission. The identification of neonatal infection by real-time PCR targeting SARS-CoV-2 in nasopharyngeal or oropharynx swabs is still the gold standard [40]. This test was defined by taking into account that the respiratory tract is the main site of SARS-CoV-2 infection in adults and, thus, contains a higher viral load. In neonates, however, main SARS-CoV-2 infection and transmission sites have not been identified. Another method measures the concentration of IgM in fetal serum. Although it has been shown feasible in detecting circulating viral load, it has important limitations because of its large cross-reactivity and false positives, with the results also depending on physiological immunological changes occurring in the newborn [40]. Therefore, a histopathological evaluation of the placenta or tissue viral detection could be more successful in determining if SARS-CoV-2 transplacental transmission occurred and the real impact of the viral infection on placental physiology. For this purpose, immunohistochemistry, *in situ* hybridization and RNA scope are currently proposed to identify and confirm the presence of SARS-CoV-2 in fetal cells of the placenta, mainly in syncytiotrophoblasts (STBs) and Hoffbauer cells [40].

Indeed, viral particles of SARS-CoV-2 have been identified in the STB layer, placental cotyledon and placental submembrane [22,41,42]. Placentas from COVID-19 positive mothers also show higher fibrin deposition, signs of inflammation and lesions consistent with maternal and fetal vascular malperfusion, independent of evidence of vertical transmission [22,43,44]. Furthermore, placental intervillous inflammatory infiltrate from COVID-19 patients presents neutrophils, monocytes and macrophages expressing activation markers [45]. Taken together, these findings underline a transmission susceptibility, even though the mechanisms are still unclear and certainly not manifested in all COVID-19 positive pregnancies.

**Figure 2:**
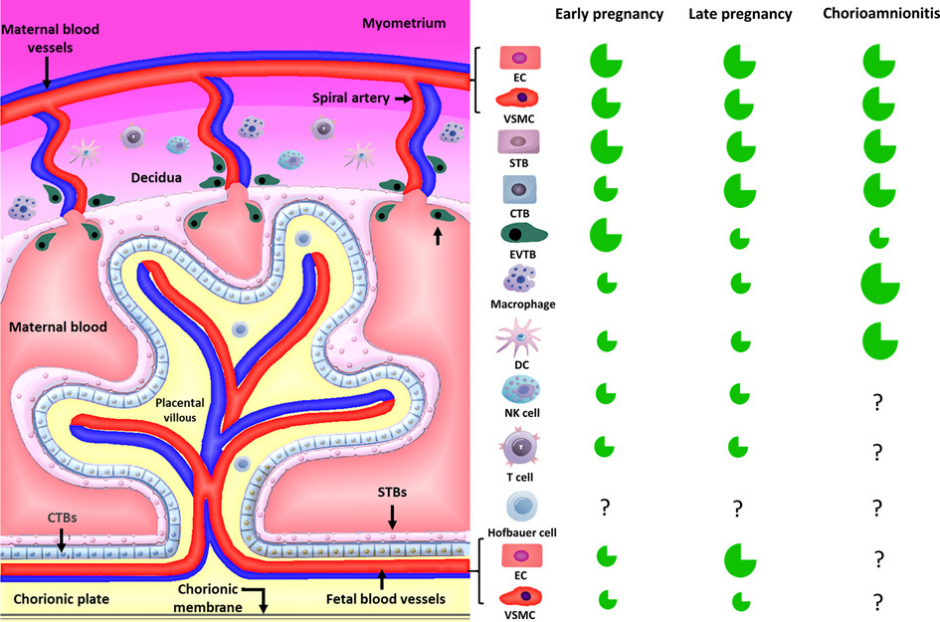
Placental villous structure and cells present in the uteroplacental unit Fetal blood vessels are separated from maternal blood by the feto–maternal interface composed by a layer of CTBs and another of STBs. Vessels from both sides are composed by vascular smooth muscle cells (VSMCs) and endothelial cells (ECs). The vessels containing arterial blood are presented in red, and the vessels containing venous blood are in blue. The fetal side (space in yellow) has the chorionic plate and the chorionic membranes; Hofbauer cells are macrophages of fetal origin. The extravillous trophoblasts (EVTBs) are invasive trophoblasts responsible for remodeling maternal spiral arteries, allowing increased blood flow in the villous. The decidua on the maternal side of the placenta have a large quantity of immune cells, such as macrophages, natural killer (NK) cells, lymphocytes T (T cells) and dendritic cells (DCs). The decidua are the modified portion of the endometrium developed after the implantation as a result of intense remodeling and immune cell recruitment. On the right, a scheme shows the dynamic expression of ACE2 protein (in green) in each placental cell type during early pregnancy, late pregnancy and chorioamnionitis.

It is noteworthy that the extensive expression of ACE2 in the uteroplacental unit throughout gestation also supports a higher placental susceptibility to SARS-CoV-2 infection, potentially enabling vertical transmission. Placental ACE2 expression is illustrated in Figure 2. Another important aspect is the spatial-temporal distribution of ACE2 in placentas during pregnancy. In early pregnancy, ACE2 is more concentrated in the decidua, in the area surrounding the villi, whereas a low content of ACE2 is found in the feto–maternal interface (Figure 2A) [20]. As gestation advances, the ACE2 protein expression pattern changes and higher expressions are found in STBs, villous endothelial cells and cytotrophoblasts (CTBs), as shown in Figure 2B [20–22]. These changes in ACE2 distribution suggest that the placental susceptibility to SARS-CoV-2 and the disease manifestation may vary throughout gestation.

The co-expression of ACE2 with TMPRSS2 and other proteins essential for SARS-CoV-2 cell entry has been recently described in the uteroplacental unit at different gestational ages. [23] However, most of these studies rely only on RNA sequencing of frozen placental samples; and they lack additional information on the levels of ACE2 expression at the membrane, as a receptor, and on enzymatic activity in the placenta. They also diverge about the period in pregnancy when there is a higher expression of ACE2 in the feto–maternal interface, but they agree that high levels of ACE2 are present in all gestational ages [23,24]. In addition, there is a consensus that the co-expression of ACE2 and TMPRSS2 in the placenta and chorioamniotic membrane is negligible, suggesting that the infection of the placenta by SARS-CoV-2 is unlikely through this route, independent of gestational age [22–24]. High concentrations of a desintegrin and metalloproteinase (ADAM) 17 (ADAM17), however, a key metalloproteinase cleaving ACE2 from cell membranes, have also been described in placentas, which could confer additional protection against SARS-CoV-2 infection by enhancing ACE2 shedding and reducing its availability in the plasma membranes [23].

Alternative entry routes for SARS-CoV-2 independent of TMPRSS2 priming are emerging and also may be considered for placental infection [23,45,46]. Cathepsin L and furin are largely expressed in placentas at all gestational ages. Even though they themselves do not provide sufficient S priming, these proteases may assist in alternative entry routes [23]. It has recently been shown that CD147 (Figure 1B), a transmembrane glycoprotein, can also bind to S, being considered a novel entry receptor for SARS-CoV-2 [46]. CD147 is highly expressed in placentas and choroamniotic membranes [23].

Furthermore, a large number of different immune cells infiltrate the feto–maternal interface while exerting key roles in placentation and during labor [45]. Placentas naturally present a high content of maternal and fetal macrophages (Figure 2) [47], and maternal peripheral immune cells also express ACE2 [45]. In addition, macrophages express CD169, an adhesion molecule also reported to enhance SARS-CoV-2 infection through mechanisms not yet well understood [23]. In chorioamnionitis, for example, there is a high influx of macrophages and other immune cells into the placenta, and they seem to contribute to stimulate ACE2 gene expression [45]. Likewise, ACE2 is more expressed in the STB layer in placentas with chorioamnionitis in contrast with placentas of healthy pregnancies [45]. These data support the postulate that immune cells could also act as an alternative transport route for SARS-CoV-2 inside the placenta, a process potentially enhanced during infectious and inflammatory conditions such as chorioamnionitis (Figure 2).

An alternative strand of reasoning is whether SARS-CoV-2 binding can reduce ACE2 bioavailability in placentas and in the feto–maternal circulation, thus blunting ACE2’s essential and protective biological effects [25,48,49]. This is important because ACE2 depletion has been reported in placentas infected by SARS-CoV-2 [22,50]. These placentas also present a phenotype similar to that observed in preeclampsia and IUGR [27,51–53].

Infected STBs have also been shown to exhibit increased expression of Ang II receptor type 1 (AT1) and the anti-angiogenesis factor fms-like tyrosine kinase-1 (s-Flt1), with decreased expression of the pro-angiogenic placental growth factor (PlGF) and Ang II receptor type 2 (AT2) [50]. In line with these findings, higher levels of sFlt-1 and AT1 autoantibodies, both markers of preeclampsia, were observed in COVID-19 positive mothers [50] and in cases of patients developing preeclampsia-like syndrome among women with severe COVID-19 manifestation [53].

To understand the possible impact that a significant ACE2 depletion could cause on placental development and on maternal hemodynamics, in the next sessions we review ACE2’s main physiological roles and biological functions during pregnancy.

## ACE2 as an integrative component of physiological systems in pregnancy

**Figure 3:**
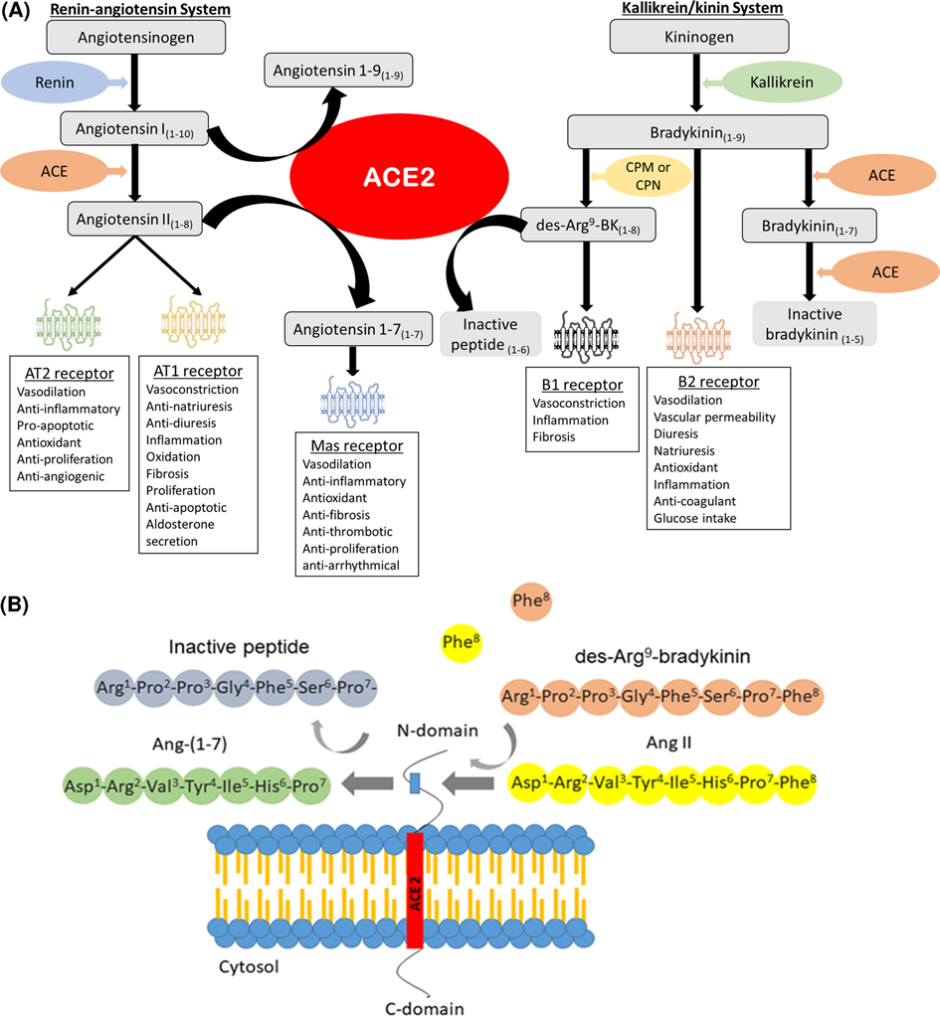
Physiologic system regulated by ACE2 (**A**) Overview of the RAS and KKS having ACE2 as a central component cleaving mainly Ang II, but also Ang I, and inactivating des-Arg^9^BK. (**B**) Illustration of the ACE2 catalytic site releasing a single residue (Phe) from its substrates Ang II and des-Arg^9^-BK. Abbreviations: BK, bradykinin; B1, BK receptor type 1; B2, BK receptor type 2; CPM, carboxypeptidase M; CPN, carboxypeptidase N.

The RAS is a major physiological system regulating blood pressure and volume. This system has been extensively studied since the discovery of the first component, renin, in experimental models of hypertension [54–56]. Briefly, renin is an aspartyl protease that acts on the circulating angiotensinogen to generate Ang I, which is subsequently cleaved by ACE, generating Ang II [57,58] (Figure 3A). Ang II’s effects include a potent vasopressor response when binding to its receptor AT1, which is abundantly and widely distributed in the human tissues [58,59]. Moreover, Ang II has pro-inflammatory, pro-oxidant, pro-angiogenic and anti-apoptotic effects through AT1 receptor activation [56,60]. Finally, Ang II stimulates the release of aldosterone by the adrenal glands and of vasopressin by neurohypophysis, promoting the reabsorption of sodium and water in the kidneys [58,60,61]. The Ang II receptor AT2 has opposite effects to AT1, stimulating vasodilatation, apoptosis, antioxidant defences, anti-proliferative and anti-angiogenic responses [59,60]. However, AT2 is less abundant in adult tissues, being expressed more in the reproductive organs and in fetal tissues [62,63].

ACE2 is a zinc metallopeptidase inhibited by EDTA; it was discovered almost 50 years after the discovery of its homolog ACE [19,64]. The N-domain and the catalytic site of ACE2 are in the extracellular surface of this enzyme, which contains the His‐Glu‐Met‐Gly‐His zinc‐binding motif that allows the cleavage of circulating peptides (Figure 3B). The C-domain is near the hydrophobic region that anchors the enzyme in the cell membrane [19,65,66].

The main ACE2 product is Ang 1-7, an antagonist of the deleterious effects of Ang II and AT1. When ACE2 cleaves Ang II to form Ang 1-7, this peptide binds to the G-protein coupled receptor Mas and promotes vasodilation, anti-inflammatory, anti-remodeling, anti-arrhythmical and anti-proliferative effects [19,67,68]. Therefore, the ACE2/Ang 1-7/Mas receptor axis has sparked interest as the counterregulatory axis of RAS [69,70].

Although ACE2 activity is generally linked to RAS, ACE2 can also degrade kinins, playing a critical role in regulating the KKS [66,71]. The KKS participates in the pathophysiology of hypertension, thrombosis, diabetes and pregnancy disorders [72–75]. This system is described in Figure 3A. Briefly, an inactive protein precursor called kininogen is cleaved by kallikreins (tecidual and plasmatic), releasing kinins [76,77]. Bradykinin (BK) is the main kinin of this system and has a potent vasodilator action [77–79]. It can be degraded by ACE, forming inactive metabolites, or cleaved by carboxypeptidases M and N, forming the active pro-inflammatory des-Arg^9^-BK and des-Arg^10^-Lys-BK [80,81].

There are two known BK receptors, kinin receptor type 1 (B1) and type 2 (B2). B2 is widely distributed in tissues under physiological conditions, while B1 is generally less expressed and seems to be induced during inflammatory processes [72,82]. BK has more affinity to B2. Its activation mediates vasodilatation, increased vascular permeability, increased renal blood flow and high intake of glucose by tissues [83,84]. In contrast, des-Arg^9^-BK is the main kinin actively binding to B1, and it is involved in the pathogenesis of inflammatory diseases [72,82]. It is interesting to note that ACE2 cannot degrade BK as ACE does. However, ACE2 is an alternative metabolic pathway to inactivate des-Arg^9^BK [69].

The ability of ACE2 to cleave des-Arg^9^BK and Ang II, both being pro-inflammatory agents, suggests the ACE2/Ang 1-7/Mas axis as an anti-inflammatory pathway by counteracting the effects of these two peptides.

## ACE2’s role in the maternal hemodynamics

The RAS contributes to maintaining and adapting the maternal hemodynamics during pregnancy through its direct action on cardiovascular and renal systems. This is an important developmental step during pregnancy to ensure an adequate uterine blood flow and feto–maternal exchange. Disruptions in this process may lead to complications in pregnancy and ultimately increase feto–maternal mortality [85].

An increase in plasma renin activity (PRA) occurs mainly in the third trimester in healthy pregnancies [86]. A simultaneous increase in angiotensinogen production by the liver is stimulated by estrogens, resulting in elevated levels of Ang I. In addition, augmented levels of Ang II can be observed despite the reduction in serum ACE activity [86]. These findings suggest an activation of alternative RAS pathways enhancing Ang II production in the maternal circulation during pregnancy. Similarly, aldosterone levels gradually increase proportionally to estrogens and Ang II, promoting water and sodium retention and increasing blood volume [87]. Further, plasmatic levels of ACE2 and Ang 1-7 are significantly augmented during pregnancy [30,86]. This is probably caused by the fact that estrogen can also up-regulate ACE2 [88].

In healthy pregnancies, the activation of RAS contributes to a progressive increase in plasma volume, which can reach up to 40% at term [89]. Further, there is a dramatic elevation in the maternal cardiac output reaching up to 50%. This increase is stimulated to enhance the perfusion of the uterus and kidneys. As a result, the glomerular filtration rate proportionally increases, reaching up to 50%, and the kidneys increase in size [90,91].

Interestingly, despite the overactivation of RAS and increase in circulating Ang II, a reduction in the total peripheral resistance [86] and a slight reduction in blood pressure, particularly in the second trimester [89], are observed. Additionally, the heart and kidneys are exposed to a massive increment in workload without causing organ damage. Yet, even with elevated levels of Ang II and aldosterone, pregnancy is associated with increased diuresis [85,90,91]. These paradoxes are still considered intriguing and not well understood, but they suggest a very refined balance between both axes of the RAS or between RAS and KKS.

Hormonal regulation and physiological adaptations are, however, dramatically disrupted in hypertensive disorders of pregnancy and other severe pregnancy complications. In preeclampsia, for example, the most common and severe hypertensive disorder of pregnancy [92], PRA, Ang I, Ang II, Ang 1-7, ACE2 and aldosterone levels are significantly reduced in comparison to normal pregnancy, while ACE serum activity is slightly augmented [30,86,87]. Reduced production of estrogen by the placenta in preeclamptic pregnancies is also observed and can trigger the subsequent alterations [93].

It has been observed that women with healthy pregnancies and delivering at term have a diminished vascular response to the vasopressor effects of Ang II when compared with non-pregnant women or preeclamptic women [94]. Possible explanations for this event include an increased production of prostaglandin E2 (PGE2) and nitric oxide (NO), both potent vasodilators [26,95]. Further, KKS is activated and urinary kallikrein is increased in normotensive pregnancies [26]; thus, there is also an enhanced vascular response to BK vasodilator effects in healthy pregnancies [96,97]. Alternatively, the highest prevalence of a monomeric form of AT1, which is less sensitive to Ang II, may also contribute to reducing vascular sensitivity to Ang II [98]. All these alternatives are increased by the counterregulatory action of Ang 1–7, since its level is substantially increased in non-complicated gestations [86] and associated with increased vasodilation in mesenteric arteries in rats [99].

Taking all these aspects into account, the ACE2/Ang 1-7/Mas axis may consist in a central point regulating maternal hemodynamic alterations in healthy pregnancies, as it relates directly or indirectly to all the mechanisms mentioned above. Furthermore, ACE2 has been described as regulating other associated mechanisms that contribute to increasing vasodilation, such as the down-regulation of AT1 in vascular smooth muscle cells (VSMCs), the inhibition of ACE in arteries and the activation of AT2-mediated vasodilatation, mainly through a synergic action with Ang 1-7 and Mas receptor actions [100,101].

Another important aspect of ACE2 in pregnancy is that it may confer protection to cardiac and renal tissues while the essential cardiac and hemodynamic adaptations take place [18,102,103]. ACE2 has been shown to be cardioprotective in several models of cardiovascular diseases and prevents renal fibrosis and inflammation, as observed in models of Alport syndrome and diabetic nephropathy [18,102–105]. ACE2 is extensively expressed in the heart vasculature and its genetic knockout in mice can cause high blood pressure, augmented expression of hypoxia genes in the cardiac tissue and cardiac dysfunction [104]. Pregnant ACE2 knockout mice exhibit higher blood pressure and are more susceptible to Ang II effects, even though other cardiovascular parameters evaluated, such as heart weight and cardiac output, are not affected [27,29]. However, to the best of our knowledge, to date there are no data reporting maternal ACE2 cardiac expression changes during pregnancy or whether significant ACE2 depletion could cause severe maternal cardiac dysfunction during pregnancy or postpartum.

On the other hand, ACE2 spatial-temporal expression in the kidneys during pregnancy has been well described. In the renal proximal and distal tubule cells of rats, ACE2 and Ang 1-7 were co-expressed and their levels progressively increased throughout pregnancy [106]. Such changes were accompanied by an augmentation of ACE2 activity at the end of gestation [107]. In the reduced uterine perfusion pressure (RUPP) model, on the other hand, diminished Ang 1–7 staining in the kidneys was observed in contrast with normal pregnancy, as were increased protein/creatinine ratios and mild glomerular lesions, including hypercellularity and variable lobulation [107]. However, despite an absence of Ang 1-7 in cells not expressing ACE2, there was no significant difference in Ang 1–7 levels between RUPP and control [107]. These findings support the view that ACE2/Ang 1-7/Mas up-regulation, either systemically or in the kidneys, may be important in determining a healthy pregnancy.

Further investigations are needed to better understand the exact function of the ACE2/Ang 1-7/Mas axis in pregnancy and maternal hemodynamics. This is important because hypertensive disorders of pregnancy, such as preeclampsia and gestational diabetes, are highly associated with increased cardiovascular risks and severe chronic renal disorders after pregnancy [108,109].

## ACE2 in placentation

Placentation is an overly complex process involving fetal cells, maternal uterine circulatory changes and immunomodulation [110–112]. In the first stages, trophoblasts differentiate, proliferate and invade the maternal decidua. These processes are followed by a refined orchestration of the remodeling of uterine vessels to provide an adequate blood flow and perfusion to the uteroplacental unit and to the fetus [111,112]. At mid-pregnancy, the placenta is almost completely formed, and angiogenesis processes in the feto–placental unit prevail along with the maintenance of vasodilation of the main uteroplacental arteries. Both mechanisms ensure an adequate blood perfusion and nutrient exchange between fetal and maternal circulations [110,113].

Most components of the RAS and KKS, as well as prostaglandins and NO, have been described in the uteroplacental unit of humans, hinting at a complex regulation of these systems during placental development. ACE2 and Ang 1–7 are expressed in STBs, CTBs, endothelial cells of villous blood vessels, VSMCs of primary villi, syncytial and decidua [20,114]. Prorenin, (pro)renin receptor, AT1 and AT2 proteins are also expressed throughout gestation in the feto–maternal interface (STBs and CTBs) and in invasive trophoblasts such as the extravillous trophoblasts (EVTBs), while ACE is concentrated in the fetal endothelial cells [21,115]. Kallikrein and B2 are similarly expressed in STBs, EVTBs, chorionic villi and fetal endothelial cells [26]. Information on the des-Arg^9^BK/B1 axis is scarce, but B1 receptors have been detected in trophoblasts from the first trimester of pregnancy [75]. Finally, endothelium nitric oxide synthase (eNOS) expression has also been detected in STBs, CTBs, EVTBs and the fetal endothelium [20,26].

Considering the significant presence of Ang II/AT1, ACE2/Ang 1–7, BK/B2, prostaglandins and NO in trophoblasts, endothelial cells and VSMCs of placentas, the dominant axis may vary significantly depending on the gestation time and the different locations of the uteroplacental unit. Such variations also seem to be dynamic and differently modulated in healthy and complicated pregnancies.

Shallow trophoblast invasion of the decidua and blunted angiogenesis are related to spontaneous miscarriages and to the early onset of hypoperfusion in the placenta, a phenotype observed in gestational hypertension, IUGR and preeclampsia [116]. Ang II is described as stimulating migration and invasion of EVTBs in first trimester placentas [115,117]. Similarly, renin and AT1 mRNA levels are higher in placentas from 10 to 14 weeks (elective interruption) than in term placentas [21]. Additionally, AT1 is located mainly in STBs in early pregnancy [115]. Further, Ang II binding to AT1 can stimulate vascular endothelial growth factor (VEGF) secretion, contributing to enhanced angiogenesis [118].

The KKS also regulates trophoblast invasion and angiogenesis. BK stimulates migration of trophoblasts through its receptor B2, having the same effect on endothelial cells and endothelial progenitor cells. It also stimulates metalloproteinases (MMP) such as MMP9, which is active in the remodeling of uterine arteries by trophoblasts [75]. PGE2 is also reported to mediate migration in human trophoblasts [119].

Conversely, an extensive and exaggerated trophoblast invasion into the myometrium can lead to another clinical condition named placenta accrete. This condition is characterized by the adhesion of the placenta directly to the myometrium fibers, resulting in severe maternal hemorrhage [114]. Therefore, the pro-invasive effects of Ang II/AT1 and BK/B2 should be well regulated and constantly refined, mainly by ACE2.

Indeed, expression of kallikrein and eNOS are markedly increased in EVTBs of placenta accrete, and B2 is augmented in fetal endothelial cells [26]. Ang 1–7 has been shown to inhibit proliferation and angiogenesis in VSMCs and in several types of cancer cells, even though in renal carcinoma it promoted migration and invasion of endothelial cells [114]. These effects were not evaluated in trophoblasts and placental endothelial cells particularly. Nonetheless, in placentas from spontaneous miscarriages, Ang 1–7 is significantly augmented in the syncytium, suggesting that an excessive up-regulation of Ang 1–7 could compromise implantation and invasion, creating a predisposition to miscarriage [20]. These findings indicate that exaggerated ACE2 activity and Ang 1–7 production could have anti-angiogenic and anti-proliferative properties in the uteroplacental unit, potentially counterregulating other RAS and KKS components that promote proliferative and angiogenic responses.

As gestation progresses from midterm to term, trophoblast invasion ceases, and the placenta is already well anchored. The uterine arteries are also remodeled at this point and become more sensitive to vasoactive factors [29]. Angiogenesis, however, is still required mainly on the fetal side, and a progressive increase in the vascularization of both the maternal and the fetal sides of the placenta is observed. Additionally, from this moment on, uterine blood flow increases progressively and is regulated mainly by vasoactive factors [120]. In guinea pigs, which display similar ACE2 and Ang 1–7 distribution to that of human placentas, ACE2 and Ang 1–7 were shown to be highly expressed in invasive trophoblasts surrounding the spiral arteries and in VSMCs of these arteries. They were also colocalized with kallikrein, B2 receptor and VEGF [114]. Thus, the ACE2/Ang 1–7 axis may possibly constitute an important component regulating the tonus of the main uterine arteries.

A dynamic distribution of AT1 protein expression throughout pregnancy is also observed predominating mainly in STBs in early pregnancy and in endothelial villous vessels at term [115]. These dynamic changes may be crucial to modulate the intensity and location of main Ang II effects. They are also associated with alterations in expression of AT2 receptors as well as of ACE2.

In placentas of women with hypertensive disorders of pregnancy, this dynamic regulation of Ang II receptors and ACE2 may be blunted. It was previously shown in placentas of hypertensive pregnancies delivering at term that AT1 expression was still higher in STBs in late gestation, and that this correlated with hypertension severity [121]. In chorionic villi of placentas from preeclampsia pregnancies, levels of Ang II and AT1 mRNA expression were also higher [122]. In rats, AT2 mRNA expression dramatically increased in the uterine artery from day 18 to term, and the blockage of AT2 resulted in reduced uterine arterial blood flow and increased resistance of uterine arteries [120].

In normal pregnancies, the ACE protein is more importantly expressed in fetal endothelial cells, and this expression progressively increases as the fetus grows [21]. This pattern of ACE expression and localization may favor increased production of Ang II in placental vessels from the fetal side, where angiogenesis is still occurring and is essential to maintain fetal perfusion [21]. However, a local vasoconstriction in response to Ang II should be simultaneously inhibited. It is therefore suggested that ACE2 and Ang 1–7 could play an important role in this regulation, as they are broadly expressed in the uteroplacental unit and can directly inhibit Ang II while stimulating prostaglandins and NO synthesis, favoring a sustained vasodilation in the feto–placental vessels. As well, ACE2 is abundantly expressed in STBs [20,21], which are cells in close contact with the maternal blood. The presence of ACE2 in this location suggests a potential contribution of this enzyme to increasing maternal circulating levels of Ang 1–7, thus helping to reduce maternal systemic vascular resistance [21,86].

However, whether ACE2 is an essential or only a coadjutant mechanism regulating maternal vascular resistance and placentation in humans is still unclear. Previous studies in full-term placentas from normal and preeclamptic pregnancies did not identify differences in ACE2/Ang 1–7 immunostaining or mRNA expression [20,122]. However, mRNA expression of the Mas receptor showed a decrease in chorionic villi in preeclampsia when compared with normal pregnancies [122].

Animal models, which allow more extensive and detailed investigations, support the view that ACE2 deficiency is associated with placental dysfunction, IUGR and a preeclampsia-like phenotype. ACE2 knockout models have been used to identify ACE2 deficiency effects on pregnancy. Importantly, ACE knockouts do not make pregnancies inviable, but a phenotype of IUGR was indicated, as gestational body weight gain was reduced in this model [27,29]. In addition, this model shows reduced pup weight and length, and fetal death is higher compared with control mice. Blood pressure is also higher in ACE2 knockout mice and maintained during pregnancy, probably reflecting RAS imbalance. The levels of Ang 1–7 are decreased in the plasma and placenta, while Ang II levels are 2.5-fold higher than in wildtype mice despite the fact that placental ACE activity is reduced [27]. Such findings indicate that ACE2 is possibly the main regulator of Ang II levels in the placenta throughout pregnancy.

Yamaleyeva et al. [29] did not observe changes in trophoblast invasion in ACE2 knockout mice when assessing it by immunostaining. However, decreased pup-to-placental weight ratio, an indication of placental insufficiency, was observed. Further, ACE2 knockout mice presented higher levels of hypoxia markers in the trophospongium and labyrinth zone [29]. Interestingly, vascular dysfunction was also observed. Uterine arteries from ACE2 knockout mice had greater maximal response to phenylephrine and were more sensitive to Ang II effects. AT2 protein expression, on the other hand, was decreased, and a tendency for collagen deposition was observed. Finally, in the umbilical artery, peak systolic velocity and resistance index were lower, indicating a higher vascular resistance and reduced perfusion to the fetus [29].

In line with findings in ACE2 knockouts, another model of IUGR induced by the administration of glucocorticoids during pregnancy showed ACE2 alterations. Expression of ACE2 mRNA in the basal zone at 19 and 21 days of pregnancy was higher in the IUGR group compared with controls [28]. However, in the labyrinth zone the ACE2 mRNA expression was dramatically increased from day 19 to 21 in the control group; but in the IUGR group, ACE2 mRNA levels remained constant and lower than the control group at day 21. In addition, ACE2 protein expression and Ang 1–7 levels were decreased in the labyrinth zone in these IUGR rats at day 21, while Mas protein expression was up-regulated [28], indicating a possible feedback regulation. Additionally, in pregnancy-induced hypertension using the RUPP model, reduced levels of Ang 1–7 in the uterus and placenta were observed, and ACE2 mRNA concentration was reduced in the uterus when compared with the controls [123].

Collectively, a large body of evidence supports an important role for ACE2 on placental development and vascularization; and this is reinforced by experimental studies demonstrating in models of ACE2 depletion that a significant placental dysfunction can occur and be associated with risks of severe complications of pregnancy. Still, further clinical and experimental studies are needed to confirm the main downstream mechanisms primarily affected when ACE2 is depleted in the uteroplacental unit.

## Soluble ACE2 in pregnancy

**Figure 4:**
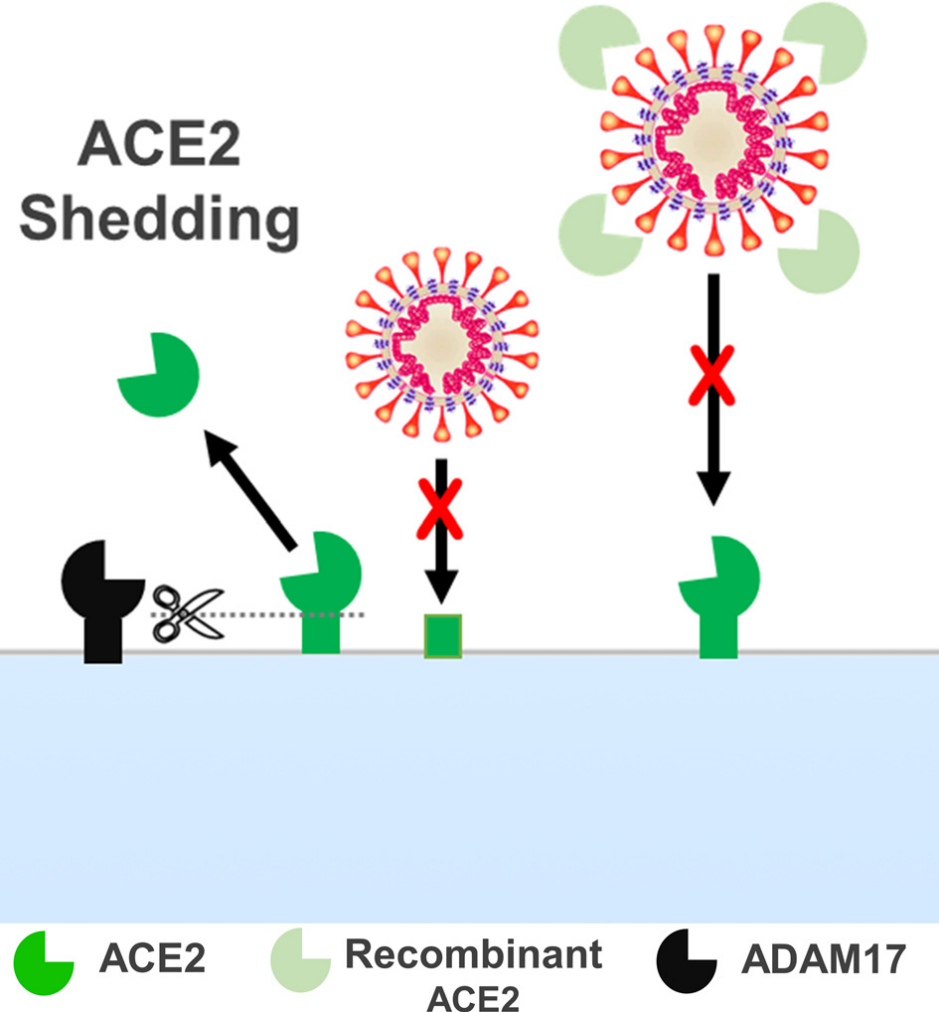
ACE2 shedding by ADAM17 and potential therapeutic effect of human recombinant ACE2 by preventing the biding of SARS-CoV-2 with transmembrane ACE2 proteins

Cell membrane-bound proteins commonly undergo a shedding process in which the membrane-bound sequence can be cleaved, releasing the soluble form of the protein, which may exert a distinct function [124]. Therefore, ACE2 is susceptible to the cleaving process. ACE2 can be found as a full-length protein anchored in the cell membrane or as a circulating ectodomain (soluble form) [125,126]. Studies have demonstrated that ADAM17 is the main metalloproteinase responsible for ACE2 shedding (Figure 4) [126,127].

Higher levels of soluble ACE2 could be beneficial to prevent COVID-19, as SARS-CoV-2 would bind to the circulating ACE2 instead of the transmembrane. On this hypothesis, soluble human recombinant ACE2 has been proposed as a therapy for COVID-19. This is supported by *in vitro* studies demonstrating that soluble human recombinant ACE2 can reduce the SARS-CoV-2 viral load 1000–5000-fold [128,129]. However, the full-length transmembrane ACE2 corresponds to 96–98% of the total amount of ACE2 even when shedding is enhanced [130]. Thus, it is unlikely that such small variations could confer enough protection against SARS-CoV-2 or define the severity of the disease.

Nevertheless, considering that ACE2 is localized mainly in placental endothelial cells and STB [20,123], the shedding of placental ACE2 and the release of soluble ACE2 into the maternal blood could have a significant impact. Indeed, in normal pregnancies, plasmatic levels of ACE2 are higher and correlate with increased circulating ACE2 activity and enhanced levels of circulating Ang 1-7 [30]. Thus, even if the amount of soluble ACE2 corresponds to a small percentage of the total ACE2 available [130], as the levels of the enzyme substantially increase in the kidneys and the placenta during pregnancy [20,107], it is expected that the amount of soluble ACE2 follows and increases in response.

In contrast, an imbalance of ACE2 expression in tissues and shedding may deplete the physiological paracrine and intracrine effects of ACE2 in the placenta. In line with this, soluble ACE2 is indicated as a marker for cardiovascular and non-controlled diabetes diseases [131–133]. It was previously demonstrated by Patel et al. [134] that Ang II binding to AT1 can increase ADAM17 expression followed by an increase in soluble ACE2, suggesting a role of Ang II/AT1 on ACE2 shedding regulation. Hence, a deep investigation of this process in the uteroplacental unit could contribute to a better understanding of the complex processes of ACE2 regulation in pregnancy and, therefore, fill some major knowledge gaps.

## New insights of ACE2 in the pathophysiology of COVID-19 during pregnancy

Despite solid evidence that ACE2 plays a significant role in pregnancy by regulating fetal–maternal hemodynamics and placentation processes, mechanisms regulating and mediating ACE2 expression and function in pregnancy are still unknown. Because we now see an increased number of COVID-19 pregnancies and an association with the severity of COVID-19 disease, more studies are emerging that investigate maternal and placental ACE2 expression and activity changes and potential mechanisms related to it and the pathophysiology of COVID-19. A main concern is about ACE2 acting as an entry door for the virus in the placenta and fetal circulation, therefore promoting or facilitating intrauterine vertical transmission. However, little attention has been given to a secondary impact that SARS-CoV-2 can have on feto–maternal circulation and placentation by causing significant ACE2 depletion.

**Figure 5:**
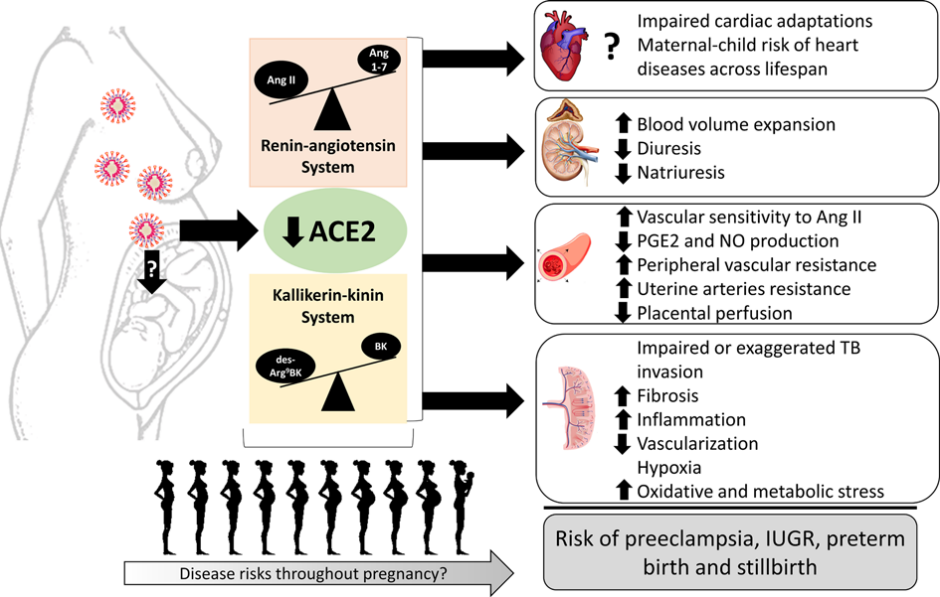
Summary of the consequences of ACE2 depletion resulting from SARS-CoV-2 infection in pregnancy on maternal and fetal health and disease risks across lifespan Abbreviation: TB, trophoblast.

The interruption of ACE2 physiological function could be a main factor contributing to increased risks of complications and disease in mother and child exposed to COVID-19 during pregnancy. This postulate is summarized in Figure 5 and supported by recent findings pointing to the fact that SARS-CoV-2 infection during pregnancy contributes to increasing the severity of COVID-19 and rates of adverse outcomes, including preterm birth and preeclampsia [8,12,13,135]. Other complications observed in association with COVID-19 were small for gestational age, IUGR, admission to neonatal ICU and stillbirth [8–10,12,38]. A preeclampsia-like syndrome was recently reported in pregnant women with COVID-19 [53]. Lower levels of ACE2 protein in placentas from COVID-19 positive pregnancies have also been reported [22], hinting that SARS-CoV-2 infection may directly or indirectly change ACE2 expression and biological functions in the placenta as well as in maternal and fetal circulations. In addition, potential therapeutic strategies preventing the binding of SARS-CoV-2 and ACE2 either by blocking the RBD of the viral S-protein or by using competitive recombinant ACE2, as well as by using ACE2-derived peptides, should be considered and tested to prevent and treat pregnancy complications resulting from COVID-19 infection.

COVID-19 is strongly associated with coagulopathy, endothelial dysfunction and vasculitis [136]. It is postulated that SARS-CoV-2 promotes endothelial dysfunction through ACE2 depletion by activating both RAS and KKS and their downstream pro-fibrotic, pro-inflammatory, pro-oxidative and pro-thrombosis mechanisms in vessels [137,138]. In fact, hamsters treated with the protein S developed lung damage [139]. Levels of phospho-AMP-activated protein kinase (pAMPK), phospho-ACE2 and ACE2 were decreased in the endothelium of vessels from damaged lungs, while murine double minute 2 (MDM2) was up-regulated. Both pAMPK and MDM2 can regulate ACE2 expression by phosphorylation and ubiquitination, respectively. These alterations were combined with mitochondrial fragmentation [139]. Further *in vitro* studies confirmed that S protein promotes ACE2 depletion, and that this contributes, at least in part, to impaired mitochondrial function, impaired eNOS activity and, ultimately, to endothelial dysfunction [139].

Similarly, COVID-19 was shown to promote endothelial dysfunction in placentas, particularly in pregnancies more severely affected by COVID-19. Placentas from women with mild and severe COVID-19 have been shown to express higher levels of von Willebrand factor (vWf) with reduced levels of claudin-5 and VE-cadherin in the decidua and chorionic villi, revealing the presence of thrombosis, infarcts and vascular wall remodeling, but mainly in women with severe COVID-19 [140].

Autoimmune responses may play a role on regulating ACE2 in COVID-19 pregnancies. The binding of soluble ACE2 with SARS-CoV-2 can induce a series of conformational alterations in both proteins that may result in the exposure of new epitopes [141]. It has been shown that the SARS-COV-2/ACE2 complex when recognized by antigen presenting cells can stimulate the production of autoantibodies targeting ACE2 [141]. If confirmed, this mechanism could explain the higher risk of patients with diseases known to induce higher levels of soluble ACE2, such as hypertension, heart disease and diabetes [132,141].

To date, ACE2 autoantibodies have not been evaluated in pregnancies with COVID-19. However, it is important to consider this kind of investigation because the mechanism of autoantibody formation appears to depend directly on the circulating levels of soluble ACE2, which is known to increase during pregnancy [51]. In addition, recombinant soluble ACE2 has been proposed as a therapy for COVID-19, for it is known to form stable complexes with SARS-CoV-2 [128]. Nevertheless, no report has described whether recombinant ACE2 can also stimulate an autoimmune response against transmembrane ACE2; and this should certainly be considered in the evaluation of this therapy’s effectiveness, particularly in pregnant patients.

## Maternal and fetal outcomes of COVID-19

Substantial data on COVID-19's impact in pregnancy have been recently released. There are still conflicting reports; and some methodological limitations have been addressed, such as COVID-19 testing only in symptomatic pregnancies and at delivery time, a lack of control for confounder factors (such as age, comorbidities, multiparity etc.), limited comparisons with non-pregnant women, and an underrepresentation of pregnancies diagnosed with COVID-19 in early pregnancy [12,142]. Therefore, we here summarize the most concise data reported until June 2021.

The most common symptoms reported in COVID-19 pregnant patients were fever (34–84%), cough (28–71%), dyspnea (18–40%) and myalgia (16–38%) [7,9,10,12,13]. Pregnant women were less likely to present fever, myalgia and dyspnea when compared with non-pregnant women [12,13]. However, fever and shortness of breath of any duration among pregnant women with COVID-19 were associated with increased risk for severe maternal and fetal complications [142]. Common laboratory findings included lymphopenia (22–35%), increased C-reactive protein or procalcitonin (23–54%) and elevated transaminase (16%) [9,10,12]. When universal testing was applied, a significant percentage (44–82%) of pregnant women who tested positive for SARS-CoV-2 were asymptomatic [12,142].

Among women of reproductive age (15–44 years) who tested positive for COVID-19, the need for hospitalization was more likely for pregnant (31%) than non-pregnant (5%) women; but these data make no distinction between pregnancy-related hospitalization and COVID-19-related hospitalization [13]. Admission to the ICU was also more frequent in pregnant women diagnosed with COVID-19 than in pregnant women without the disease or even non-pregnant women with COVID-19 [12,13,143]. Pregnant women with COVID-19 stayed 3.73 days longer in the ICU than women without COVID-19 [142]. Furthermore, diagnosed pregnant women were more likely to require mechanical ventilation and extracorporeal membrane oxygenation than non-pregnant women with COVID-19 [12,13]. Risk factors for disease severity among pregnant women include advancing age, obesity, non-white ethnicity and diabetes [8,10,12]. In Brazil, higher incidences of hypertension and obesity were observed among pregnant women who died from COVID-19 compared with pregnant women who recovered [144].

The maternal mortality rate from COVID-19 was reported to be low (<1%) [9,10,12,13]; however, when compared with pregnant women without COVID-19, the disease increased the odds of maternal death [12]. These findings have been reproduced by others. In another large cohort from the United States including more than 40000 pregnancies and 6380 with COVID-19, rates of in-hospital deaths, myocardial infarction and venous thromboembolism were higher in pregnancies with COVID-19 [145]. In this study, the odds of preeclampsia (1.21 with 95% CI 1.11–1.33) and preterm birth (1.17 with 95% CI 1.06–1.29) were considerably higher among pregnancies with COVID-19 than in those without COVID-19 [145]. Moreover, there are disparities in mortality rates among countries [142], with Brazil presenting the highest mortality rate among pregnant women with COVID-19 [144]. Possible explanations for such disparities include the high prevalence of hypertensive disorders, mainly preeclampsia, and obesity in pregnant women, as well as socioeconomic issues and deficiencies in the healthcare system [144].

Preterm birth was the most common pregnancy complication reported to affect 9–39% of all COVID-19 positive pregnancies [8–10,12,38]. COVID-19 increased the risk of both medically advised and spontaneous preterm birth [142]. Even though most of these cases were medically advised, an approximate rate of 6% of preterm births occurred spontaneously [10,12]. Other maternal–fetal complications reported in pregnant women with COVID-19 included admission to neonatal ICU (15%), IUGR (9%), fetal distress (8.8%), miscarriage (2%), stillbirth (0.7%) and neonatal death (0.5%) [9,12]. Indeed, recent meta-analyses comparing these outcomes between pregnancies with and without the disease showed that COVID-19 increased the risk of admission to neonatal ICU, stillbirth and lower birth weight [12,143].

Finally, SARS-CoV-2 infection during pregnancy was shown to increase the likelihood of gestational hypertension and preeclampsia, even among asymptomatic women [8,135,142,143]. The severity of the disease was also shown to contribute significantly to increased rates of adverse fetal outcomes (mainly preterm birth and low birth weight) and risks of preeclampsia and gestational diabetes [142,143].

## Conclusion and future perspectives

This review describes the main physiological roles of ACE2 in different important aspects of placental development and maternal hemodynamic regulation during pregnancy. The correlation between ACE2 receptor function and placental dysfunction is well established in the preeclampsia and IUGR literature. However, the correlation in the clinical context of a pregnancy complicated by COVID-19 is less clear. Yet, several knowledge gaps exist in defining the exact role of ACE2 in pregnancy. Filling these gaps may dramatically enhance the understanding of the real impact of COVID-19 in pregnancy.

Evidence that SARS-CoV-2’s binding and internalization can deplete transmembrane ACE2 suggests a worse response to the disease. This dilemma can also extend to COVID-19 in pregnancy. This is supported by a large placental expression of ACE2 and its co-expression with proteases required for SARS-CoV-2 cell entry, even though this colocalization is questioned and apparently varies between individuals. The elucidation of this gap is important and can help elucidate individual susceptibility as well as risks of disease severity and the likelihood of intrauterine vertical transmission.

To date, it is well known that placentas are susceptible and can be infected by SARS-CoV-2. It is also known that in these cases, placental ACE2 is down-regulated, which can potentially contribute to altering key physiological processes during placental development and vascularization. This is supported by the fact that a preeclampsia-like syndrome can emerge in cases of COVID-19 infection during pregnancy. However, the gestational age at exposure may also be an important factor determining placental vascular responses or outcomes.

In order to accelerate the investigation of these conditions involving ACE2, the simulation of placental explants and organoids should be used. These models can create a placental unit composed of the main cells targeted by SARS-CoV-2 and the observation of changes in essential placentation mechanisms resulting from ACE2 depletion and the inhibition of its main downstream pathways. This is important because access to human placentas is extremely limited, particularly at the early gestational stages.

Regardless of these limitations, investigations of clinical correlations between placental vascular function, the ACE2 receptor and COVID-19 infection remain equally important in the understanding of potential effects of infection on the fetus, placental function and inflammatory and vascular stress, as they may have long-term effects not yet discovered in mother and child.

## Abbreviations

ACE: angiotensin-converting enzyme
ADAM: a desintegrin and metalloproteinase
Ang: angiotensin
AT1: angiotensin II receptor of type 1
AT2: angiotensin II receptor of type 2
B1: kinin receptor of type 1
B2: kinin receptor of type 2
BK: bradykinin
CoV: coronavirus
COVID-19: coronavirus disease 2019
CTB: cytotrophoblast
eNOS: endothelium nitric oxide synthase
EVTB: extravillous trophoblast
IUGR: intrauterine growth restriction
KKS: kallikrein–kinin system
MERS: Middle East Respiratory Syndrome
NO: nitric oxide
PGE2: prostaglandin E2
PRA: plasma renin activity
RAS: renin–angiotensin system
RBD: receptor-binding domain
RUPP: reduced uterine perfusion pressure
S: Spike protein
SARS: severe acute respiratory syndrome
STB: syncytiotrophoblast
TMPRSS2: transmembrane serine protease 2
VEGF: vascular endothelial growth factor
VSMC: vascular smooth muscle cell
